# Syndrome d'Apert chez un congolais de 60 ans: à propos d'une observation

**DOI:** 10.11604/pamj.2015.20.433.6742

**Published:** 2015-04-30

**Authors:** Léon Kabamba Ngombe, Christophe Mwamba Kabamba, David Kakez Nday, Jimmy Ngoie Fundi, Tony Kayembe Kitenge, Luboya Numbi

**Affiliations:** 1 Université de Kamina, Faculté de Médecine, Département de Santé Publique, Unité de toxicologie, République Démocratique du Congo; 2 Université de Lubumbashi, Faculté de Médecine, Département de Santé Publique, Unité de toxicologie, République Démocratique du Congo; 3 Zone de Santé de Dilolo, hopital General de Dilole, République Démocratique du Congo; 4 Zone de Santé de Kolwezi, Hôpital General de Kolwezi, Kolwezi, République Démocratique du Congo; 5 Université de Lubumbashi, Faculté de Médecine, Département de Pédiatrie, République Démocratique du Congo

**Keywords:** Craniosténoses, syndactylie bilatérale, syndrome d′apert, adulte, congolais, craniosynostosis, bilateral syndactyly, Apert syndrome, adult, Congolese

## Abstract

Le syndrome d'Apert est une rare acrocéphalosyndactylie caractérisée par une dysmorphie crânio-faciale avec une crâniosténose, une syndactylie aux mains et aux pieds et d'autres malformations cérébrales. La coexistence de plusieurs malformations avec un important lot de préjudices esthétiques constitue la gravité de ce syndrome. Une prise en charge précoce et multidisciplinaire s'avère important. Les auteurs rapportent une observation rare d'un syndrome d'apert chez un patient congolais âgé de 60 ans qui n'a jamais bénéficié d'une prise en charge. Ainsi, cette observation décrit les aspects cliniques et évolutifs de cette affection.

## Introduction

Le syndrome d'Apert est une rare acrocéphalosyndactylie caractérisée par une dysmorphie crânio-faciale avec une crâniosténose, une syndactylie aux mains et aux pieds, et d'autres malformations cérébrales [1]. Ce syndrome a été décrit pour la première fois par Dr Apert en 1906 [2]. C'est une affection rare dont la transmission se fait le plus souvent selon un mode autosomique dominant bien que des cas sporadiques existent [3, 4]. Cette malformation touche particulièrement les sujets de race caucasienne, asiatique et afro-américaine [5]. Dans notre milieu, il n'y a pas des données relatives à cette malformation. Le but de ce travail est de décrire cette malformation chez un patient âgé de 60 ans, congolais de race noire qui n'a jamais bénéficié d'un quelconque traitement dès son bas âge, et mettre l'accent sur les aspects cliniques et évolutifs de cette affection.

## Patient et observation

**Figure 1:**
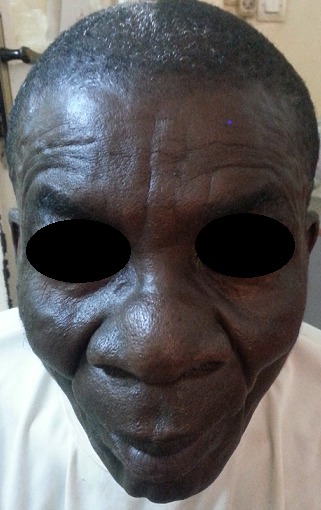
Une dysmorphie craniofaciale

**Figure 2:**
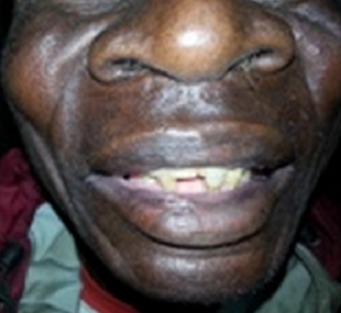
Une dysmorphie craniofaciale

**Figure 3:**
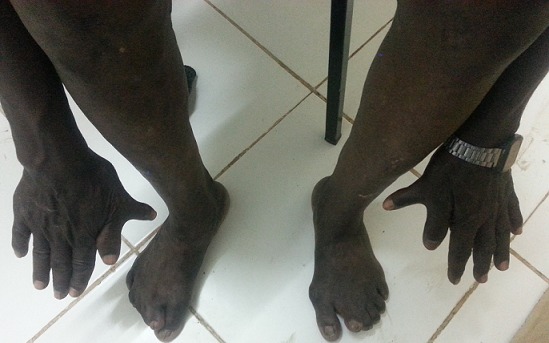
Une syndactylie bilatérales des mains et pieds

Le patient était de sexe masculin, âgé de 60 ans avec un poids de 58 Kg. Son état nutritionnel était bon (IMC 23). A l'admission le motif de consultation était des douleurs abdominales accompagnées d'une fièvre sans horaire depuis 5 jours. Par ailleurs, le patient se plaignait également des éruptions cutanées prurigineuses au niveau des membres inférieurs qui n'ont pas constitué le motif de son admission à l'hôpital. Dans les antécédents, il est issu d'un mariage non consanguin, sans notion des malformations congénitales dans sa famille mais une de ses filles avait une syndactylie au niveau du pieds droit. L'examen physique révèle une dysmorphie craniofaciale modifiée probablement par l’âge du patient (Figure 1, Figure 2), une malposition dentaire, des éruptions cutanées sous forme des macules au niveau des faces antérieures des jambes et des pieds, une syndactylie bilatérales et symétriques des mains (pré-axiale) (Figure 3) et des pieds (Figure 3). Au vue des signes présentés par le patient, nous avons pensé à un syndrome d'apert probable associé à un paludisme chez une personne âgée de race noire. Les examens de laboratoire ont révélé une goutte épaisse positive, Hb: 13g/dl, le GB: 7000 /mm^3^, VS: 10 mm/h, glycémie: 78 mg/dl. Un traitement fait des antalgiques, d'un antipaludéen, d'une pommade antimycosique avait été instauré. Un bilan plus poussé de sa malformation n'a pas été effectué par manque des moyens financiers.

## Discussion

Le syndrome d'Apert a été décrit pour la première fois en 1906 [2]. Il est caractérisé par la triade: craniosténose, sévère syndactylie des mains et des pieds, une dysmorphie faciale [1, 2]. Selon la littérature, il n'existe pas de prédominance de sexe et la prévalence est de 1 pour 100.000 naissances en France, 15 pour 100.000 naissances aux USA [6]. En ce qui concerne notre observation, le patient est de sexe masculin, âgé de 60 ans, présentant une dysmorphie faciale (occiput aplati, un gros nez avec une base aplati, malposition dentaire, aspect de retrognatisme), une syndactylie bilatérale et symétrique aux quatre membres, hypo pigmentation des faces antérieures des jambes et des pieds sous forme des macules; Ces signes illustrent les anomalies cliniques évocatrices du syndrome d'Apert, et l’âge du patient constitue la particularité de notre observation. Car il y a peu ou presque pas d'observation du syndrome d'Apert décrit chez la personne âgée, mais un cas de Syndrome d'apert chez une fille âgé de 31 ans a été décrit par ELAFRIT [7] en Tunisie. Il est connu que la dysmorphie est présente dès la naissance, avec un tableau clinique très polyvalent qui comporte des malformations cranio-faciales en rapport avec une brachycéphalie, un aplatissement de l'occiput, un bombement frontal antérieur et une hypoplasie de l’étage moyen de la face avec un rétrécissement des loges orbitaires à l'origine d'un proptosis et parfois un exorbitisme, une exophtalmie, un hypertélorisme, un ptosis, un nez mince et pointu, un rétrognatisme, une hypoplasie des voies aériennes supérieures et de l'ethmoïde, et une fente palatine [8, 9]. Dans notre cas, la dysmorphie est non classique probablement modifié par l’âgé du patient. En effet, l'existence d'une syndactylie cutanée et/ou osseuse des deux mains et pieds (aspect en moufle des extrémités), de larges phalanges distales du pouce et du gros orteil et un pouce court avec clinodactylie radiale [1, 10] chez tout patient avec d'acrocéphalosyndactylie de type 1 excluent le diagnostic du syndrome de Crouzon. Ainsi, La présence d'une syndactylie osseuse pré-axiale du pouce des deux mains, et une syndactylie des deux pieds avec fusion touchant les 2^ème^ et 3^ème^ doigts, nous a poussé à exclure le syndrome de Crouzon [1, 10]. Par ailleurs, les manifestations oculaires sont très fréquentes et complexes. Il s'agit d'une exophtalmie, un hypertélorisme et un strabisme. L'altération de la fonction visuelle est la complication la plus sévère en rapport avec une kératite d'exposition, des cicatrices cornéennes, une amblyopie ou une atrophie optique [11]. Chez notre patient, il n'y a pas eu des plaintes en rapport avec l'altération de la fonction visuelle mais un aspect exophtalmique non franc des yeux a été constaté. D'autres anomalies existent notamment: du système nerveux central [12], cardiaques et viscérales [13]. Quant à notre patient, il a présenté l'hypopigmentation de la peau et des cheveux; Et ceci correspond aux anomalies décrites par COHEN [14]. L'origine génétique du syndrome d'Apert est actuellement certaine, et la transmission se fait sur un mode autosomique dominant. L'existence des cas sporadiques suggère le rôle de néo-mutations génétiques non héréditaires qui seraient favorisées par un âge paternel élevé [15]. Ceci n'est pas le cas dans notre observation.

Cependant, dans le syndrome d'Apert, il existe une activation du facteur de croissance fibroblastique type 2 (FGFR2) par mutation du gène codant son récepteur. Ceci a pour conséquence une augmentation du métabolisme osseux et un trouble de la synthèse osseuse. Deux types de mutations sont décrites: la S252w dans 83% des cas et la P253r dans 37% des cas [16]. La mutation S252w est plus fréquente chez les patients présentant une fente labiale tandis qu'avec la mutation P25r le degré de syndactylie est plus sévère. Ceci pourrait expliquer la syndactylie des mains et des pieds de notre patient. Pour BENMILOUD [17], l’évolution d'un enfant atteint du syndrome d'Apert dépend de l'environnement familial, d'une prise en charge précoce de la crâniosténose et de la présence ou l'absence de malformations cérébrales. De ce fait, la prise en charge nécessite une collaboration pluridisciplinaire afin d’établir un calendrier thérapeutique qui tenant compte des différentes anomalies observées. Par contre, l'absence de la compression cérébrale et des anomalies cardio-respiratoires sont des éléments de bon pronostic concernant la survie de l'enfant surtout dans nos milieux sous développés avec un plateau technique très limité. Notre patient illustre cela car il a su atteindre 60 ans. Le patient n'a bénéficié d'aucune intervention, et il s'avère moins important d'en faire à son âge.

## Conclusion

Le syndrome d'Apert est une affection rare qui touche particulièrement les sujets de race caucasienne, asiatique et afro-américaine. Le diagnostic clinique est basé sur la triade: craniosténose, sévère syndactylie des mains et des pieds, une dysmorphie faciale. La gravité réside dans la coexistence de plusieurs malformations avec un important lot de préjudices esthétiques. Ainsi, l'association à des anomalies viscérales aggrave le pronostic vital et fonctionnel. Une prise en charge précoce multidisciplinaire avec la collaboration entre pédiatres, neurochirurgiens et ophtalmologistes s'avère important pour le patient.
